# Supplementary material for: Suppression of Ventilation-Induced Diaphragm Fibrosis through the Phosphoinositide 3-Kinase-γ in a Murine Bleomycin-Induced Acute Lung Injury Model
Source: Int J Mol Sci. 2024 Jun 8;25(12):6370. doi: 10.3390/ijms25126370 (PMC11203512; doi:10.3390/ijms25126370)
Supplement: Supplementary file 1 [file ijms-25-06370-s001.zip › ijms-2991431-supplementary.pdf]

Article

Supplementary Materials: Suppression of Ventilation-Induced Diaphragm Fibrosis through the Phosphoinositide 3-Kinase-γ in a Murine Bleomycin-Induced Acute Lung Injury Model

Li-Fu Li [1,2,3,4], Chung-Chieh Yu [1,2,3,4], Chih-Yu Huang [1,2,3,4], Huang-Pin Wu [1,2,3], Chien-Ming Chu [1,2,3], Ping-Chi Liu [1,2,4] and Yung-Yang Liu [5,6,7,*]

[1] Division of Pulmonary and Critical Care Medicine, Department of Internal Medicine, Chang Gung
Memorial Hospital, Keelung 20401, Taiwan; lfp3434@cgmh.org.tw (L.-F.L.); ycc@cgmh.org.tw (C.-C.Y.); hcu121@yahoo.com.tw (C.-Y.H.); whanpyng@cgmh.org.tw (H.-P.W.); rocephen@cgmh.org.tw (C.-M.C.); ewind14@cgmh.org.tw (P.-C.L.)

[2] Department of Internal Medicine, Chang Gung University, Taoyuan 33302, Taiwan

[3] Department of Respiratory Therapy, Chang Gung Memorial Hospital, Keelung 20401, Taiwan

[4] Community Medicine Research Center, Chang Gung Memorial Hospital, Keelung 20401, Taiwan

[5] Chest Department, Taipei Veterans General Hospital, Taipei 112201, Taiwan

[6] School of Medicine, Faculty of Medicine, National Yang Ming Chiao Tung University, Taipei 112304, Taiwan

[7] Institute of Clinical Medicine, School of Medicine, National Yang Ming Chiao Tung University, Taipei 112304, Taiwan

* Correspondence: yyliu1103@gmail.com; Tel.: +886-2-28712121 (ext. 3071); Fax: +886-2-28757858

Table S1. Physiologic conditions at the beginning and end of ventilation.

	Nonventilated	Nonventilated with bleomycin	V_T 6 ml/kg with bleomycin	V_T 10 ml/kg with bleomycin	V_T 10 ml/kg with bleomycin, AS605240	V_T 10 ml/kg with bleomycin, $PI3K\gamma^{-/-}$
PH	7.41±0.07	7.38±0.05	7.33±0.09	7.38±0.05	7.39±0.07	7.36±0.08
PaO2 (mmHg)	98.4±0.3	96.1±0.3	85.8±0.3*	76.7±2.6*	84.9±2.1*	86.5±1.8*
PaCO2 (mmHg)	39.1±0.4	39.8±0.2	40.3±1.2	36.8±1.2	37.8±1.4	37.4±1.2
MAP (mmHg)						
Start	85.4±1.2	83.5±0.4	84.7±1.1	81.7±2.5	83.8±2.2	84.2±1.9
End	85.0±0.4	80.7±0.2	79.2±2.1*	75.1±2.4*	78.7±1.9*	79.8±2.1*
PIP (mmHg)						
Start			16.0±1.2	16.7±1.3	16.3±1.1	16.2±1.1
End			17.0±1.4	17.9±1.5	17.5±1.4	17.3±1.4

At the end of the study period, we obtained data of mean arterial pressure and arterial blood gases from the nonventilated control mice and mice ventilated at a tidal volume of 6 ml/kg or 10 ml/kg for 8 h (n = 10 per group). The normovolemic statuses of mice were maintained by monitoring mean artery pressure. Data are presented as means ± SDs. * Indicates that $P < 0.05$ when compared to the nonventilated control mice with bleomycin pretreatment. MAP = mean arterial pressure; $PI3K\gamma^{-/-}$ = phosphoinositide 3-kinase-γ-deficient mice; PIP = peak inspiratory pressure; V_T = tidal volume.

Supplementary methods

Bleomycin Administration

Bleomycin, which acts by preventing incorporation of thymidine into the DNA, promotes EMT by inducing DNA strand breaks [1,2]. The mice received a single dosage of 0.075 units of bleomycin in 100 μl of sterile normal saline (2 mg/kg, Sigma, St. Louis, MO, USA) intratracheally for 5 days. Bleomycin exposure results in an acute inflammatory reaction followed by pulmonary fibrosis that slowly resolves

[1,2].

Pharmacological Inhibitors

PI3K-γ inhibitor (AS605240, Sigma, St. Louis, MO) 5 mg/kg was given intraperitoneally 1h before MV based on our present and previous studies that showed 5 mg/kg inhibited PI3K-γ activity [3].

Measurement of Inflammatory Cytokines

Active TGF-β1 with a lower detection limit of 4.61 pg/ml were measured in BAL fluid using a commercially available immunoassay kit containing primary polyclonal anti-mouse antibodies that were cross-reactive with rat and mouse TGF-β1 (Biosource International, Camarillo, CA, USA). Each sample was run in duplicate according to the manufacturer's instructions.

Immunoblot Analysis

The diaphragm was homogenized in 0.5 ml of lysis buffer as previously described [1,2]. Crude cell lysates were matched for protein concentration, resolved on a 10% bis-acrylamide gel, and electrotransferred to Immobilon-P membranes (Millipore Corp., Bedford, MA, USA). For the assay of caspase-3, calpain, atrogin-1, MuRF-1, PGC-1α, PI3K-γ, and glyceraldehydes-phosphate dehydrogenase (GAPDH), Western blot analyses were performed with respective antibodies (New England BioLabs, Beverly, MA, USA, Santa Cruz Biotechnology, Santa Cruz, CA, USA, and Novus

Biologicals, Littleton, CO, USA). Blots were developed by enhanced chemiluminescence (NEN Life Science Products, Boston, MA, USA).

Masson's Trichrome Stain and Fibrosis Area

The diaphragm from control, nonventilated mice, mice exposed to MV for 8 h while breathing room air were paraffin embedded, sliced at 4 μm, deparaffinized, stained sequentially with Weigert's iron hematoxylin solution, Biebrish scarlet-acid fuchsin solution, and aniline blue solution according to the manufacturer's instruction of a trichrome kit (Sigma, St. Louis, MO, USA). A blue signal indicated positive staining of collagen. The diaphragmatic blue-stained fibrotic area in Masson's trichrome stained slice was analyzed using NIH image 1.6 software. Average number of 5 nonoverlapping fields in Masson's trichrome staining of paraffin lung sections, 6 mice per group, was analyzed for each section by a single investigator blinded to the mouse genotype [4].

Real-Time Polymerase Chain Reaction

For isolating total RNA, the diaphragms were homogenized in TRIzol reagents (Invitrogen Corporation, Carlsbad, CA) according to the manufacturer's instructions. Total RNA (1 μg) was reverse transcribed by using a GeneAmp PCR system 9600 (PerkinElmer, Life Sciences, Inc., Boston, MA), as previously described[5]. The following primers were used for real-time PCR: PI3K-γ, forward primer 5'-

GCCCCGGGTAGGTCTAGATT- 3’ and reverse primer 5’-CATGCCCTATGCGACCTGA -3’; and GAPDH as internal control, forward primer 5’-AATGCATCCTGCACCACCAA-3’ and reverse primer 5’-GTAGCCATATTCATTGTCATA-3’ (Integrated DNA Technologies, Inc., Coralville, IA)[6]. All quantitative PCR reactions using SYBR Master Mix were performed on a CFX96 Touch Real-Time PCR Detection system (Bio-Rad Laboratories, Inc., Hercules, CA, USA). All PCR reactions were performed in duplicate and heated to 95°C for 5 min followed by 40 cycles of denaturation at 95°C for 10 sec, and annealing at 55°C for 30 sec. The relative gene expression was calculated using $2^{-\Delta\Delta CT}$ method and the standard curves (cycle threshold values versus template concentration) were prepared for each target gene and for the internal control (GAPDH) in each sample. The specific gene’s cycle threshold (Ct) values were normalized to the GAPDH and compared with the nonventilated control group with bleomycin that was assigned a value of 1 to calculate the relative fold change in expression.

References

1. Li, L.F.; Liu, Y.Y.; Kao, K.C.; Wu, C.T.; Chang, C.H.; Hung, C.H.; Yang, C.T. Mechanical ventilation augments bleomycin-induced epithelial-mesenchymal transition through the Src pathway. *Lab. Invest.* **2014**, *94*, 1017-1029.

2. Li, L.F.; Kao, K.C.; Liu, Y.Y.; Lin, C.W.; Chen, N.H.; Lee, C.S.; Wang, C.W.; Yang, C.T. Nintedanib reduces ventilation-augmented bleomycin-induced epithelial-mesenchymal transition and lung fibrosis through suppression of the Src pathway. *J. Cell. Mol. Med.* **2017**, *21*, 2937-2949.
3. Wei, X.; Han, J.; Chen, Z.Z.; Qi, B.W.; Wang, G.C.; Ma, Y.H.; Zheng, H.; Luo, Y.F.; Wei, Y.Q.; Chen, L.J. A phosphoinositide 3-kinase-gamma inhibitor, AS605240 prevents bleomycin-induced pulmonary fibrosis in rats. *Biochem. Biophys. Res. Commun.* **2010**, *397*, 311-317.
4. Yang, R.; Jia, Q.; Li, Y.; Mehmood, S. Protective effect of exogenous hydrogen sulfide on diaphragm muscle fibrosis in streptozotocin-induced diabetic rats. *Exp. Biol. Med (Maywood).* **2020**, *245*, 1280-1289.
5. Liu, Y.Y.; Chen, N.H.; Chang, C.H.; Lin, S.W.; Kao, K.C.; Hu, H.C.; Chang, G.J.; Li, L.F. Ethyl pyruvate attenuates ventilation-induced diaphragm dysfunction through high-mobility group box-1 in a murine endotoxaemia model. *J. Cell. Mol. Med.* **2019**, *23*, 5679-5691
6. Wu, Y.X.; Han, X.; Chen, C.; Zou, L.X.; Dong, Z.C.; Zhang, Y.L.; Li, H.H. Time series gene expression profiling and temporal regulatory pathway analysis of angiotensin II induced atrial fibrillation in mice. *Front. Physiol.* **2019**, *10*, 597.
